# Supplementary material for: Are “Free From” Foods Risk-Free? Lactose-Free Milk Fermentation Modulates Normal Colon in a Gut Microbiota in Vitro Model
Source: Microorganisms. 2025 Aug 29;13(9):2021. doi: 10.3390/microorganisms13092021 (PMC12471900; doi:10.3390/microorganisms13092021)
Supplement: Supplementary file 1 [file microorganisms-13-02021-s001.zip › microorganisms-3811396-supplementary.pdf]

Supplementary tables for:

Are "Free From" Foods Risk-Free? Lactose-Free Milk Fermentation Modulates Normal Colon in a Gut Microbiota In Vitro Model.

Lorenzo Nissen[1,2], Flavia Casciano[1], Alessandra Bordoni[1,2] and Andrea Gianotti[1,2,*].

[1]DiSTAL - Department of Agricultural and Food Sciences, *Alma Mater Studiorum* –

University of Bologna, P.za G. Goidanich, 60, 47521 Cesena, Italy.

[2]CIRI - Interdepartmental Centre of Agri-Food Industrial Research, *Alma Mater Studiorum*

– University of Bologna, P.za G. Goidanich, 60, 47521 Cesena, Italy.

Table S1. Primers pairs employed for PCR and qPCR reactions and quantifications.

Table S2. MANOVA categorical descriptors for the volatilome, categorized for the matrix.

Table S3. MANOVA categorical descriptors for the volatilome, categorized for the time.

Table S4. Baseline values of beneficial VOCs in mM.

Table S5. Baseline values of detrimental VOCs in mM.

Table S6. Fold change and baseline abundance at the phylum level by Metataxonomy analysis of colonic microbiota *in vitro* fermentation.

Table S7. Fold change and Baseline abundance at the family level by Metataxonomy analysis of colonic microbiota *in vitro* fermentation.

Table S8. Fold change and Baseline abundance at the species level by Metataxonomy analysis of colonic microbiota *in vitro* fermentation.

Table S9. Venn diagram exclusive species.

Table S10. Venn diagram occurrence of species.

Table S11. qPCR absolute quantifications and shifts over time of selected bacterial taxa.

Table S1. Primers pairs employed for PCR and qPCR reactions and quantifications.

Bacterial taxa	Target	Sequence 3′-5′	Bp	MT*	Reference	Standard curve R^2
Eubacteria	*V3-V4 16S*	Eub518-R: ATTACCGCGGCTGCTGG<br>Eub338-F: ACTCCTACGGGAGGCAG	147	57.6<br>63.5	[55]	0.996
Firmicutes	*V3-V4 16S*	Firm934-F: GGAGYATGTGGTTTAATT<br>Eub338-R: ACTCCTACGGGAGGCAG	300	60.5<br>63.5	[56]	0.992
Bacteroidetes	*V3-V4 16S*	Bact934-F: GGARCATGTGGTTTAATT<br>Bact1060-R: AGCTGACGACAACCATG	250	58.9<br>59.4	[56]	0.996
Lactobacillales	*V3-V4 16S*	F-Lac: GCAGCAGTAGGGAATCT<br>R-Lac: GCATTYCACCGCTACACA	340	59.8<br>58.3	[57]	0.993
Bifidobacteriaceae	*RecA*	RecA-F: CGTYTCBCAGCCGGAYA<br>RecA-R: CCARVGCRCCGGTCATC	220	60.3<br>59.2	[58]	0.997
Enterobacteriaceae	*V3-V4 16S*	Enterob-F: TGCCGTAACTTCGGGAG<br>Enterob-R:<br>TCAAGGACCAGTGTTCAG	450	64.2<br>60.3	[59]	0.979

Table S2. MANOVA categorical descriptors for the volatilome, categorized for the matrix.

VOCs ID.	% of contribution				MANOVA
	Baseline	BC	L	LF	*P value*
Ethyl Acetate	0.00	22.55	38.48	38.97	0.206198
Acetic acid	29.10	22.12	37.68	11.10	0.328800
Propanoic acid	32.84	27.01	15.69	24.46	0.085876
Propanoic acid, 2-methyl	0.00	16.39	65.48	18.13	0.759150
Butanoic acid	6.71	24.30	33.19	35.80	0.859820
Butanoic acid, 2-methyl-	0.00	22.99	52.95	24.06	0.798437
Benzoic acid, methyl ester	0.00	0.00	100.00	0.00	0.571071
Pentanoic acid	0.00	21.81	52.02	26.18	0.833455
Pentanoic acid, 3-methyl	0.00	100.00	0.00	0.00	0.605610
Hexanoic acid	2.49	4.31	68.21	24.99	0.727496
Octanoic acid	0.00	0.00	100.00	0.00	0.605610
Isopropyl Alcohol	0.00	67.28	13.47	19.25	0.041554
Ethyl alcohol	1.66	30.38	24.46	43.50	0.347908
1-Propanol	0.97[b]	15.94[ab]	48.33[a]	34.77[ab]	0.038298
1-Butanol	1.27	27.81	34.31	36.61	0.375363
1-Butanol, 3-methyl	0.00	27.90	36.79	35.31	0.377423
1-Pentanol	0.00[b]	47.22[a]	19.14[ab]	33.64[ab]	0.024339
3-Buten-1-ol, 3-methyl-	0.00	33.08	0.00	66.92	0.752188
2-Buten-1-ol, 3-methyl-	0.00	100.00	0.00	0.00	0.288082
Isotridecanol-	0.00	100.00	0.00	0.00	0.605610

1-Hexanol	0.00	47.40	22.86	29.74	0.081168
1-Heptanol	10.25	37.91	20.94	30.91	0.236838
1-Hexanol, 2-ethyl	11.11	31.51	31.46	25.92	0.498309
1-Octanol	8.32	28.66	31.51	31.50	0.214612
2-Octen-1-ol, (E)-	0.00	7.83	62.75	29.42	0.384360
1-Nonanol	19.33[c]	37.71[a]	12.80[c]	30.16[bc]	0.004246
1-Propanol, 3-(methylthio)-	0.00[b]	0.00[b]	46.21[a]	53.79[a]	0.012473
1-Undecanol	0.00[b]	100.00[a]	0.00[b]	0.00[b]	0.001465
Benzyl alcohol	0.00	36.32	20.86	42.82	0.076428
Phenethyl alcohol	0.00[b]	24.86[ab]	23.94[ab]	51.20[a]	0.041535
Phenol	2.96	79.74	12.94	4.36	0.627448
Phenol, 4-methyl	21.43	19.03	26.30	33.23	0.931528
Phenol,2,4-bis(1,1-dimethylethyl)	49.98	18.16	18.18	13.68	0.165495
2,4-Dimethyl-1-heptene	0.00	26.77	25.67	47.56	0.758039
Pyrazine	0.00	31.67	29.44	38.89	0.716937
Thiazole, 2-methyl-	0.00[a]	15.12[a]	9.57[a]	75.32[b]	0.002113
Thiazole	0.00	24.42	54.18	21.40	0.844314
Pyrazine, methyl-	0.00	56.32	12.63	31.05	0.112721
Dimethyl trisulfide	0.00	5.04	67.69	27.27	0.649985
Benzene,1,3-bis(1,1-dimethylethyl)	3.92	35.08	26.85	34.15	0.324468
Thiophene, 2-pentyl-	0.00	100.00	0.00	0.00	0.145460
2-Acetylthiazole	0.00	38.59	34.93	26.48	0.150983
Indole	8.54	33.14	19.86	38.46	0.259383
1H-Indole, 3-methyl	45.73	25.70	17.25	11.32	0.239777

[abc] Different letters indicate statistical significance by Tuckey's HSD test ($p < 0.05$).

Table S3. MANOVA categorical descriptors for the volatilome, categorized for the time.

VOCS ID	% of contribution of VOCs descriptors			MANOVA
	**0h**	**16h**	**24h**	***p value***
Ethyl Acetate	0.00	40.68	59.32	0.091955
Acetic acid	38.11	21.68	40.21	0.517651
Propanoic acid	42.31	27.34	30.35	0.393138
Propanoic acid, 2-methyl	0.00	11.66	88.34	0.426811
Butanoic acid	9.74	20.19	70.07	0.096857
Butanoic acid, 2-methyl-	0.00	15.76	84.24	0.257943
Benzoic acid, methyl ester	0.00	100.00	0.00	0.549449
Pentanoic acid	0.00	15.46	84.54	0.297531
Pentanoic acid, 3-methyl	0.00	0.00	100.00	0.604938
Hexanoic acid	3.69	1.23	95.08	0.326409
Octanoic acid	0.00	0.00	100.00	0.604938
Isopropyl Alcohol	0.00	61.62	38.38	0.531158
Ethyl alcohol	2.46	64.39	33.15	0.055222
1-Propanol	1.44	55.44	43.12	0.285683
1-Butanol	1.89[b]	63.84[a]	34.27[b]	0.006292

1-Butanol, 3-methyl	0.00	59.66	40.34	0.098459
1-Pentanol	0.00	56.45	43.55	0.176883
3-Buten-1-ol, 3-methyl-	0.00	89.02	10.98	0.581776
2-Buten-1-ol, 3-methyl-	0.00	100.00	0.00	0.714481
1-Hexanol	0.00	53.79	46.21	0.211910
1-Heptanol	14.62	50.33	35.05	0.178765
1-Hexanol, 2-ethyl	15.79[b]	52.56[a]	31.65[b]	0.005541
1-Octanol	11.99[b]	50.40[a]	37.61[a]	0.007324
2-Octen-1-ol, (E)-	0.00	80.00	20.00	0.251424
1-Nonanol	26.43	35.83	37.74	0.859861
1-Propanol, 3-(methylthio)-	0.00	43.77	56.23	0.590129
1-Undecanol	0.00	43.67	56.33	0.866596
Benzyl alcohol	0.00	44.91	55.09	0.127237
Phenethyl alcohol	0.00	54.97	45.03	0.256022
Phenol	4.37	11.10	84.53	0.585553
Phenol, 4-methyl	29.04	15.82	55.14	0.090167
Phenol, 2,4-bis(1,1-dimethylethyl)	59.98[a]	11.95[c]	28.07[b]	0.000799
2,4-Dimethyl-1-heptene	0.00[ab]	86.87[a]	13.13[b]	0.030089
Pyrazine	0.00	72.40	27.60	0.110102
Thiazole, 2-methyl-	0.00	54.98	45.02	0.673164
Thiazole	0.00[b]	100.00[a]	0.00[b]	0.002589
Pyrazine, methyl-	2.27	53.15	46.85	0.420185
Dimethyl trisulfide	0.00	19.34	80.66	0.540444
Benzene, 1,3-bis(1,1-dimethylethyl)	5.77[a]	59.50[b]	34.73[c]	0.004898
Thiophene, 2-pentyl-	0.00	90.79	9.21	0.772985
2-Acetylthiazole	0.00	50.72	49.28	0.096672
Indole	12.29	33.18	54.53	0.114502
1H-Indole, 3-methyl	55.83	15.19	29.98	0.092098

[abc] Different letters indicate statistical significance by Tuckey's HSD test ($p < 0.05$). 0h = Baseline; 18h = T1; 24h = EP.

Table S4. Baseline values of beneficial VOCs in mM.

VOCs	Baseline (mM ± SD)
Acetic acid	29.63 ± 13.22
Propanoic acid	19.46 ± 13.85
Butanoic acid	19.09 ± 0.019

Table S5. Baseline values of detrimental VOCs in mM.

VOCs	Baseline (mM ± SD)

Phenol, 4-methyl	19.89 ± 9.71
Indole	385.22 ± 140.63
1H-Indole, 3-methyl	375.03 ± 82.13

Table S6. Fold change and baseline abundance at the phylum level by Metataxonomy analysis of colonic microbiota *in vitro* fermentation.

Sample	Gene	baseMean	BaseSD	log2(F/C)	F/C	p	-log10(p)
L	*Euryarchaeota*	0.64951775	0.10874563	0.59597615	1.51149494	0.06458507	1.18986788
	Bacteria;Other	0.44831812	0.08780056	-1.1645228	0.44611179	0.07419021	1.12965339
	Actinobacteria	3.0062834	1.43712353	0.49032257	1.40475893	0.40867046	0.38862676
	Bacteroidetes	27.8108942	4.14765528	-0.4693654	0.72228224	0.15013085	0.82353004
	Firmicutes	56.3606002	6.53419097	-0.3960653	0.75992803	0.12661202	0.89752505
	Proteobacteria	5.0267311	0.23305017	2.33310527	5.03888757	8.7833E-05	4.05634448
	Synergistetes	0.10012894	0.06463004	0.02267328	1.01584006	0.97875217	0.00932726
	Tenericutes	0.03750044	0.01158627	-2.8642279	0.13733508	0.07590587	1.11972466
	Verrucomicrobia	6.52903098	3.52555045	-0.1141559	0.92392274	0.87888487	0.05606801
	OTHER	0.02411503	0.00453267				
LF	*Euryarchaeota*	0.64951775	0.10874563	-0.8067811	0.57165588	0.08853382	1.05289081
	Bacteria;Other	0.44831812	0.08780056	-1.0309269	0.48939562	0.08566171	1.06721324
	Actinobacteria	3.0062834	1.43712353	-0.142151	0.9061671	0.83241475	0.07966023
	Bacteroidetes	27.8108942	4.14765528	-0.2935908	0.81586889	0.26964509	0.56920749
	Firmicutes	56.3606002	6.53419097	-0.9373973	0.52217406	0.03707756	1.4308888
	Proteobacteria	5.0267311	0.23305017	2.84218432	7.17104973	3.7626E-05	4.42450634
	Synergistetes	0.10012894	0.06463004	-0.6117716	0.65439263	0.57932449	0.23707811
	Tenericutes	0.03750044	0.01158627	-3.6543237	0.07942166	0.06757095	1.17023997
	Verrucomicrobia	6.52903098	3.52555045	0.36367935	1.28670323	0.58223163	0.23490421
	OTHER	0.02411503	0.00453267				
BC	*Euryarchaeota*	0.64951775	0.10874563	-0.4064309	0.7544876	0.21434025	0.66889627
	*Bacteria;*Other	0.44831812	0.08780056	-1.0939772	0.46846813	0.07981903	1.09789358
	Actinobacteria	3.0062834	1.43712353	-0.6378748	0.64265892	0.45656741	0.34049509
	Bacteroidetes	27.8108942	4.14765528	-2.4877373	0.17828567	0.02126261	1.67238338
	Firmicutes	56.3606002	6.53419097	-0.4954085	0.70936079	0.09171929	1.03753932
	Proteobacteria	5.0267311	0.23305017	3.25233323	9.52905556	1.9698E-05	4.70557845
	Synergistetes	0.10012894	0.06463004	-1.162639	0.44669469	0.40392784	0.39369621
	Tenericutes	0.03750044	0.01158627	-5.2693214	0.02592843	0.0609994	1.21467444
	Verrucomicrobia	6.52903098	3.52555045	-0.8384036	0.55926208	0.42278458	0.37388086
	OTHER	0.02411503	0.00453267				

Table S7. Fold change and Baseline abundance at the family level by metataxonomy analysis of colonic microbiota *in vitro* fermentation.

Sample	Gene	baseMean	BaseSD	log2_F/C	F/C	p	-log10(p)
L	*Methanobacteriaceae*	0.64951775	0.10874563	-0.4064309	0.7544876	0.06458507	1.18986788
	Bifidobacteriaceae	3.001685	1.43474508	0.48437082	1.39897562	0.41415814	0.3828338
	Bacteroidaceae	13.7601062	0.48719936	-0.2466251	0.84286581	0.0009789	3.00926347
	Odoribacteraceae	1.17318315	0.51044752	-1.603703	0.32903135	0.1271855	0.89556241
	Porphyromonadaceae	8.07231282	1.65512075	-0.5037986	0.70524743	0.0729343	1.13706819
	Rikenellaceae	4.62874468	1.44208705	-0.9612722	0.5136038	0.06993421	1.15531036
	Enterococcaceae	0.02385246	0.00623463	8.91531722	482.811832	1.9342E-05	4.71349243
	Lactobacillaceae	0.00942213	0.00592358	-1.2765384	0.41278477	0.38145229	0.41855977
	Leuconostocaceae	0.01529076	0.00650059	-0.3901115	0.76307063	0.27571982	0.55953202
	Streptococcaceae	1.00809194	0.58009892	-0.1913487	0.87578661	0.21879393	0.65996473
	Clostridiales;Other	4.7319156	1.15937517	-1.3328149	0.39699289	0.20076436	0.69731339
	Clostridiaceae	2.35911625	0.62889336	0.71040681	1.63626545	0.25297335	0.59692522
	Lachnospiraceae	18.727219	5.95715453	-1.4149523	0.37502215	0.3165029	0.4996223
	Peptostreptococcaceae	0.12995284	0.05454125	6.4396315	86.8005029	0.00174641	2.75785303
	Ruminococcaceae	23.9868574	1.74902508	-2.1114199	0.23141914	0.00603058	2.21964127
	Veillonellaceae	3.08011937	0.67398672	-0.4332689	0.74058183	0.04648783	1.33266069
	Coriobacteriaceae	1.16570112	0.34240326	-0.2473283	0.84245508	0.79751204	0.09826275
	Alcaligenaceae	1.37298074	0.2890682	1.66053996	3.16134823	0.82946888	0.0811999
	Desulfovibrionaceae	0.92161707	0.24201487	-0.3186537	0.80181777	0.41562683	0.38129642
	Enterobacteriaceae	1.23616629	0.35675601	4.0295845	16.3314898	4.4767E-05	4.34904544
	Verrucomicrobiaceae	6.44935539	3.48836556	-0.104604	0.93006019	0.42765545	0.36890599
	OTHER	3.39296599	1.35543	-1.8815963	0.27138327		
LF	*Methanobacteriaceae*	0.64951775	0.10874563	-0.8067811	0.57165588	0.08853382	1.05289081
	Bifidobacteriaceae	3.001685	1.43474508	-0.1473221	0.90292491	0.826774	0.08261319
	Bacteroidaceae	13.7601062	0.48719936	-0.6705287	0.62827638	0.00599404	2.22228021
	Odoribacteraceae	1.17318315	0.51044752	-3.5628068	0.08462297	0.12335919	0.90882851
	Porphyromonadaceae	8.07231282	1.65512075	0.52869857	1.44262724	0.11821696	0.92732021
	Rikenellaceae	4.62874468	1.44208705	-1.0301406	0.48966243	0.18266188	0.73835207
	Enterococcaceae	0.02385246	0.00623463	7.77433076	218.930745	9.5902E-07	6.01817336
	Lactobacillaceae	0.00942213	0.00592358	-1.2465031	0.42146855	0.37676843	0.42392549
	Leuconostocaceae	0.01529076	0.00650059	-0.4856071	0.71419645	0.49685806	0.30376766
	Streptococcaceae	1.00809194	0.58009892	-0.2684731	0.83019771	0.75240873	0.12354617
	Clostridiales;Other	4.7319156	1.15937517	-2.5658141	0.16889352	0.05334502	1.27290612
	Clostridiaceae	2.35911625	0.62889336	-3.2896687	0.10226123	0.05406181	1.26710941
	Lachnospiraceae	18.727219	5.95715453	-1.7947044	0.28823064	0.11135239	0.95330046
	Peptostreptococcaceae	0.12995284	0.05454125	2.65420571	6.29499707	0.00416234	2.38066214
	Ruminococcaceae	23.9868574	1.74902508	-3.4736073	0.09002021	0.00425317	2.37128698
	Veillonellaceae	3.08011937	0.67398672	2.03138742	4.08797795	0.22137046	0.65488033
	Coriobacteriaceae	1.16570112	0.34240326	-0.7880417	0.57912965	0.08028948	1.09534138
	Alcaligenaceae	1.37298074	0.2890682	0.46732069	1.38253949	0.15601602	0.8068308

	Desulfovibrionaceae	0.92161707	0.24201487	-0.5870111	0.66572071	0.25932352	0.5861581
	Enterobacteriaceae	1.23616629	0.35675601	4.76049232	27.1050981	8.1469E-05	4.08900619
	Verrucomicrobiaceae	6.44935539	3.48836556	0.37644053	1.2981351	0.56921271	0.24472541
	OTHER	3.39296599	1.35543	-1.7486777	0.29757439		
BC	*Methanobacteriaceae*	0.64951775	0.10874563	0.59597615	1.51149494	0.21434025	0.66889627
	Bifidobacteriaceae	3.001685	1.43474508	-0.6429454	0.64040417	0.4541097	0.34283922
	Bacteroidaceae	13.7601062	0.48719936	-3.7049961	0.07668052	0.0322213	1.49185693
	Odoribacteraceae	1.17318315	0.51044752	-3.3002233	0.10151584	0.19953029	0.69999117
	Porphyromonadaceae	8.07231282	1.65512075	-1.2704177	0.41453974	0.2203636	0.65686014
	Rikenellaceae	4.62874468	1.44208705	-3.485368	0.08928935	0.19601126	0.70771899
	Enterococcaceae	0.02385246	0.00623463	5.63010626	49.5257273	1.962E-07	6.70729078
	Lactobacillaceae	0.00942213	0.00592358	0.65211706	1.57147253	0.37109704	0.43051251
	Leuconostocaceae	0.01529076	0.00650059	0.59994447	1.51565823	0.56533569	0.24769359
	Streptococcaceae	1.00809194	0.58009892	0.87302558	1.83149985	0.81624535	0.08817928
	Clostridiales;Other	4.7319156	1.15937517	-0.6808948	0.62377826	0.09468864	1.02370214
	Clostridiaceae	2.35911625	0.62889336	-0.6124088	0.65410365	0.09981288	1.00081339
	Lachnospiraceae	18.727219	5.95715453	-0.6080897	0.65606483	0.13787163	0.8605251
	Peptostreptococcaceae	0.12995284	0.05454125	3.19996688	9.18937588	1.5951E-05	4.79720263
	Ruminococcaceae	23.9868574	1.74902508	-2.0781317	0.2368209	0.00594686	2.22571254
	Veillonellaceae	3.08011937	0.67398672	1.437504	2.70851861	0.57872991	0.23752407
	Coriobacteriaceae	1.16570112	0.34240326	1.05183849	2.0731701	0.38875753	0.41032119
	Alcaligenaceae	1.37298074	0.2890682	-0.0620144	0.95792566	0.00626662	2.20296633
	Desulfovibrionaceae	0.92161707	0.24201487	-0.3554215	0.78164123	0.45290225	0.34399552
	Enterobacteriaceae	1.23616629	0.35675601	5.17860985	36.2173691	0.00023614	3.62682503
	Verrucomicrobiaceae	6.44935539	3.48836556	-0.826041	0.56407504	0.88871329	0.05123832
	OTHER	3.39296599	1.35543	-0.847531	0.555735		

Table S8. Fold change and Baseline abundance at the species level by metataxonomy analysis of colonic microbiota *in vitro* fermentation.

Sample	Gene	baseMean	BaseSD	log2_F/C	F/C	*p*	-log10(*p*)
L	*Bifidobacterium adolescentis*	2.022471	0.772228	0.152542	1.111526	0.823928	0.084111
	Bif. bifidum	0.091063	0.091063	1.812913	3.51351	0.161436	0.791999
	Bif. longum	0.885048	0.149096	0.877064	1.836634	0.050036	1.300717
	Bacteroides massiliensis	1.354571	0.320155	-2.9765	0.127053	0.071407	1.146261
	B. uniformis	0.613561	0.003994	2.382762	5.215342	0.000182	3.740041
	Enterococcus durans	0.023329	0.003885	7.063813	133.7887	4.19E-05	4.377957
	Lactococcus garvieae	0.050657	0.020519	-0.70317	0.614219	0.297955	0.525849
	L. lactis	0.03077	0.000632	0.016081	1.011209	0.000926	3.033502
	Streptococcus thermophilus	0.787482	0.318884	-0.19195	0.875423	0.223514	0.650695
	S. vestibularis	0.066246	0.033191	0.157686	1.115496	0.216675	0.664192
	Clostridium spp.	0.015442	0.004975	-0.5298	0.69265	0.28281	0.548505
	Blautia obeum	0.817088	0.06947	0.16628	1.122161	0.007733	2.111661

	Ruminococcus gnavus	0.638337	0.374872	-2.21911	0.214774	5.95E-05	4.22566
	Faecalibacterium prausnitzii	9.534171	0.390675	-2.80714	0.142879	0.002966	2.527883
	Ruminococcus spp.	10.81209	1.215909	-1.93851	0.260886	0.027692	1.557645
	R. callidus	0.65459	0.173353	-1.84804	0.27777	0.105096	0.978412
	Collinsella aerofaciens	0.394226	0.16576	0.191503	1.141953	0.042002	1.376729
	Coprobacillus cateniformis	0.018917	0.007251	-0.58166	0.668197	0.76809	0.114588
	Bilophila wadsworthia	0.278436	0.103637	1.074326	2.105738	0.175195	0.756479
	Desulfovibrio spp.	0.61398	0.052946	-2.22572	0.213792	0.01952	1.70951
	Citrobacter freundii	0.336424	0.103009	-0.1585	0.895958	0.005834	2.234045
	Escherichia spp.	0.235647	0.034403	6.329995	80.44859	3.64E-08	7.43937
	E. albertii	0.051784	0.017242	3.323084	10.00802	0.00075	3.124865
	Klebsiella spp	0.241457	0.039589	-1.20122	0.434907	0.99966	0.000148
	Akkermansia muciniphila	6.449355	2.466647	-0.1046	0.93006	0.888713	0.051238
	OTHER	62.98286		-0.11207	0.925259		
LF	*Bifidobacterium adolescentis*	2.022471	0.772228	-0.55564	0.680354	0.543803	0.264558
	Bif. bifidum	0.091063	0.091063	0.194998	1.144723	0.911722	0.040138
	Bif. longum	0.885048	0.149096	0.471401	1.386455	0.185326	0.732063
	Bacteroides massiliensis	1.354571	0.320155	-4.22425	0.053503	0.085406	1.06851
	B. uniformis	0.613561	0.003994	2.660332	6.321787	3.18E-06	5.497528
	Enterococcus durans	0.023329	0.003885	7.344848	162.5621	8.83E-08	7.054065
	Lactococcus garvieae	0.050657	0.020519	-0.54386	0.685935	0.496195	0.304348
	L. lactis	0.03077	0.000632	-0.43032	0.742096	0.682834	0.165685
	Streptococcus thermophilus	0.787482	0.318884	-0.25869	0.835847	0.814866	0.088914
	S. vestibularis	0.066246	0.033191	0.048318	1.034059	0.860223	0.065389
	Clostridium spp.	0.015442	0.004975	-2.37424	0.192879	0.495548	0.304915
	Blautia obeum	0.817088	0.06947	-0.02837	0.980525	0.339425	0.469256
	Ruminococcus gnavus	0.638337	0.374872	-2.58716	0.166413	0.366476	0.435954
	Faecalibacterium prausnitzii	9.534171	0.390675	-4.3075	0.050503	0.003033	2.518059
	Ruminococcus spp.	10.81209	1.215909	-3.59863	0.082548	0.029508	1.530062
	R. callidus	0.65459	0.173353	-1.67141	0.313946	0.142048	0.847566
	Collinsella aerofaciens	0.394226	0.16576	-0.27578	0.826005	0.797542	0.098247
	Coprobacillus cateniformis	0.018917	0.007251	3.140258	8.816816	0.531625	0.274394
	Bilophila wadsworthia	0.278436	0.103637	0.966379	1.95393	0.12367	0.907735
	Desulfovibrio spp.	0.61398	0.052946	-3.34176	0.098635	0.015665	1.805071
	Citrobacter freundii	0.336424	0.103009	-3.5466	0.085579	0.796281	0.098934
	Escherichia spp.	0.235647	0.034403	7.109418	138.0855	1.71E-07	6.76679
	E. albertii	0.051784	0.017242	3.183871	9.087421	0.001817	2.740697
	Klebsiella spp	0.241457	0.039589	-2.67815	0.156242	0.283335	0.5477
	Akkermansia muciniphila	6.449355	2.466647	0.376441	1.298135	0.569213	0.244725
	OTHER	62.98286		-0.52637	0.694301		
BC	*Bifidobacterium adolescentis*	2.022471	0.772228	-0.88796	0.540377	0.406642	0.390788
	Bif. bifidum	0.091063	0.091063	-0.33983	0.790135	0.872533	0.059218
	Bif. longum	0.885048	0.149096	-0.23764	0.848132	0.516699	0.286762

	Bacteroides massiliensis	1.354571	0.320155	-4.86909	0.034218	0.074028	1.130606
	B. uniformis	0.613561	0.003994	-1.17536	0.442775	2E-06	5.699977
	Enterococcus durans	0.023329	0.003885	4.940787	30.7132	7.53E-07	6.123065
	Lactococcus garvieae	0.050657	0.020519	0.724287	1.652084	0.571083	0.243301
	L. lactis	0.03077	0.000632	0.830566	1.778382	0.008339	2.078886
	Streptococcus thermophilus	0.787482	0.318884	0.859848	1.814848	0.759072	0.119717
	S. vestibularis	0.066246	0.033191	1.022211	2.031029	0.958409	0.018449
	Clostridium spp.	0.015442	0.004975	-1.12471	0.458595	0.162241	0.789838
	Blautia obeum	0.817088	0.06947	1.0772	2.109937	0.861091	0.064951
	Ruminococcus gnavus	0.638337	0.374872	-1.19868	0.435674	0.343967	0.463484
	Faecalibacterium prausnitzii	9.534171	0.390675	-2.90957	0.133086	0.002474	2.606597
	Ruminococcus spp.	10.81209	1.215909	-2.08337	0.235962	0.019451	1.711063
	R. callidus	0.65459	0.173353	-2.91302	0.132768	0.154046	0.81235
	Collinsella aerofaciens	0.394226	0.16576	1.719723	3.293732	0.754357	0.122423
	Coprobacillus cateniformis	0.018917	0.007251	-0.23315	0.850778	0.00319	2.496155
	Bilophila wadsworthia	0.278436	0.103637	0.91583	1.886655	0.156647	0.805079
	Desulfovibrio spp.	0.61398	0.052946	-1.74769	0.297778	0.011985	1.921362
	Citrobacter freundii	0.336424	0.103009	2.48765	5.608636	0.1226	0.911511
	Escherichia spp.	0.235647	0.034403	7.419473	171.1922	0.002252	2.647343
	E. albertii	0.051784	0.017242	3.909784	15.03012	0.271134	0.566815
	Klebsiella spp	0.241457	0.039589	-0.00027	0.999813	0.57127	0.243158
	Akkermansia muciniphila	6.449355	2.466647	-0.82604	0.564075	0.427655	0.368906
	OTHER	62.98286		-0.71967	0.607236		

Table S9. Venn diagram exclusive species.

**Sample**	**Exclusive species**
L – (LF ∪ BC)	*Proteobacteria;Other*
	Megasphaera hominis
	Citrobacter;Other
	Mollicutes;Other
	Anaerococcus spp.
	Paraprevotella spp.
	Pseudomonas azotoformans
	Lactococcus raffinolactis
	Ruminococcus;Other
	Clostridium;Other
	Faecalibacterium;Other
LF – (L ∪ BC)	*Fusobacterium gonidiaformans*
	Bacteroides sartorii
	Bacteroides fragilis
	Leuconostoc gelidum

	Bacteroides xylanisolvens
	Parabacteroides;Other
	Flavobacteriaceae;Other
	Clostridium sulfidigenes
BC – (L ∪ LF)	*Methanosphaera stadtmanae*
	Bacteroides eggerthii
	Bacteroides stercorirosoris
	Porphyromonas;Other
	Mogibacterium spp.
	Lachnospira;Other
	Ruminococcus;Other

Table S10. Venn diagram occurrence of species.

OTUs from the BIOME file	Occurrence	Presence
Archaea;Other	3	L, LF, BC
Methanobrevibacter;s__smithii	3	L, LF, BC
Bacteria;Other	3	L, LF, BC
Actinomyces;s__	3	L, LF, BC
Parascardovia;s__	3	L, LF, BC
Bifidobacterium;Other	3	L, LF, BC
Bifidobacterium;s__adolescentis	3	L, LF, BC
Bifidobacterium;s__bifidum	3	L, LF, BC
Bifidobacterium;s__longum	3	L, LF, BC
Bacteroides;Other	3	L, LF, BC
Bacteroides;s__	3	L, LF, BC
Bacteroides;s__acidifaciens	3	L, LF, BC
Bacteroides;s__cellulosilyticus	3	L, LF, BC
Bacteroides;s__finegoldii	3	L, LF, BC
Bacteroides;s__massiliensis	3	L, LF, BC
Bacteroides;s__ovatus	3	L, LF, BC
Bacteroides;s__stercoris	3	L, LF, BC
Bacteroides;s__thetaiotaomicron	3	L, LF, BC
Bacteroides;s__uniformis	3	L, LF, BC
Bacteroides;s__vulgatus	3	L, LF, BC
Butyricimonas;s__	3	L, LF, BC
Odoribacter;s__	3	L, LF, BC
Parabacteroides;s__distasonis	3	L, LF, BC
Parabacteroides;s__merdae	3	L, LF, BC
Porphyromonadaceae;Other	3	L, LF, BC
Rikenella;s__microfusus	3	L, LF, BC
Sphingobacteriaceae;Other	3	L, LF, BC
Bacteroidetes;Other	3	L, LF, BC

Gemella;s__	3	L, LF, BC
Granulicatella;s__	3	L, LF, BC
Enterococcus;Other	3	L, LF, BC
Enterococcus;s__durans	3	L, LF, BC
Lactobacillus;s__manihotivorans	3	L, LF, BC
Weissella;s__cibaria	3	L, LF, BC
Weissella;s__viridescens	3	L, LF, BC
Lactococcus;s__garvieae	3	L, LF, BC
Lactococcus;s__lactis	3	L, LF, BC
Streptococcus;Other	3	L, LF, BC
Streptococcus;s__	3	L, LF, BC
Streptococcus;s__equinus	3	L, LF, BC
Streptococcus;s__pseudopneumoniae	3	L, LF, BC
Streptococcus;s__sobrinus	3	L, LF, BC
Streptococcus;s__thermophilus	3	L, LF, BC
Streptococcus;s__vestibularis	3	L, LF, BC
Turicibacter;s__	3	L, LF, BC
Clostridia;Other	3	L, LF, BC
Clostridium;Other	3	L, LF, BC
Clostridium;s__	3	L, LF, BC
Clostridium;s__butyricum	3	L, LF, BC
Clostridium;s__perfringens	3	L, LF, BC
Finegoldia;s__	3	L, LF, BC
Mogibacterium;Other	3	L, LF, BC
Clostridiaceae;Other	3	L, LF, BC
Dehalobacterium;s__	3	L, LF, BC
Anaerofustis;s__stercorihominis	3	L, LF, BC
Eubacterium;s__limosum	3	L, LF, BC
Anaerostipes;s__	3	L, LF, BC
Blautia;Other	3	L, LF, BC
Blautia;s__	3	L, LF, BC
Blautia;s__coccoides	3	L, LF, BC
Blautia;s__obeum	3	L, LF, BC
Blautia;s__producta	3	L, LF, BC
Coprococcus;s__	3	L, LF, BC
Coprococcus;s__catus	3	L, LF, BC
Coprococcus;s__eutactus	3	L, LF, BC
Dorea;s__	3	L, LF, BC
Dorea;s__formicigenerans	3	L, LF, BC
Lachnospira;s__	3	L, LF, BC
Lachnospira;s__pectinoschiza	3	L, LF, BC
Roseburia;s__	3	L, LF, BC
Roseburia;s__faecis	3	L, LF, BC

Ruminococcus;s__	3	L, LF, BC
Ruminococcus;s__gnavus	3	L, LF, BC
Ruminococcus;s__torques	3	L, LF, BC
Shuttleworthia;s__	3	L, LF, BC
Lachnospiraceae;Other	3	L, LF, BC
Desulfotomaculum;Other	3	L, LF, BC
Clostridium;s__	3	L, LF, BC
Tepidibacter;s__	3	L, LF, BC
Peptostreptococcaceae;Other	3	L, LF, BC
Anaerofilum;s__pentosovorans	3	L, LF, BC
Anaerotruncus;s__colihominis	3	L, LF, BC
Faecalibacterium;s__	3	L, LF, BC
Faecalibacterium;s__prausnitzii	3	L, LF, BC
Oscillospira;s__	3	L, LF, BC
Ruminococcus;s__	3	L, LF, BC
Ruminococcus;s__callidus	3	L, LF, BC
Ruminococcaceae;Other	3	L, LF, BC
Acidaminococcus;s__	3	L, LF, BC
Dialister;s__invisus	3	L, LF, BC
Megasphaera;s__elsdenii	3	L, LF, BC
Phascolarctobacterium;s__	3	L, LF, BC
Veillonella;Other	3	L, LF, BC
Veillonella;s__dispar	3	L, LF, BC
Veillonella;s__parvula	3	L, LF, BC
Veillonellaceae;Other	3	L, LF, BC
Clostridiales;Other	3	L, LF, BC
Adlercreutzia;s__	3	L, LF, BC
Collinsella;Other	3	L, LF, BC
Collinsella;s__	3	L, LF, BC
Collinsella;s__aerofaciens	3	L, LF, BC
Eggerthella;s__lenta	3	L, LF, BC
Slackia;Other	3	L, LF, BC
Slackia;s__isoflavoniconvertens	3	L, LF, BC
Coriobacteriaceae;Other	3	L, LF, BC
Coprobacillus;Other	3	L, LF, BC
Coprobacillus;s__	3	L, LF, BC
Coprobacillus;s__cateniformis	3	L, LF, BC
Coprobacillaceae;Other	3	L, LF, BC
Bulleidia;s__moorei	3	L, LF, BC
Eubacterium;s__	3	L, LF, BC
Eubacterium;s__biforme	3	L, LF, BC
Eubacterium;s__dolichum	3	L, LF, BC
Holdemania;s__	3	L, LF, BC

Firmicutes;Other	3	L, LF, BC
Alphaproteobacteria;Other	3	L, LF, BC
Sutterella;s__	3	L, LF, BC
Oxalobacter;Other	3	L, LF, BC
Bilophila;s__wadsworthia	3	L, LF, BC
Desulfovibrio;Other	3	L, LF, BC
Desulfovibrio;s__	3	L, LF, BC
Citrobacter;s__	3	L, LF, BC
Citrobacter;s__freundii	3	L, LF, BC
Erwinia;Other	3	L, LF, BC
Erwinia;s__	3	L, LF, BC
Escherichia;Other	3	L, LF, BC
Escherichia;s__	3	L, LF, BC
Escherichia;s__albertii	3	L, LF, BC
Klebsiella;Other	3	L, LF, BC
Klebsiella;s__	3	L, LF, BC
Klebsiella;s__variicola	3	L, LF, BC
Trabulsiella;s__	3	L, LF, BC
Enterobacteriaceae;Other	3	L, LF, BC
Pyramidobacter;s__piscolens	3	L, LF, BC
Acholeplasma;Other	3	L, LF, BC
Pelagicoccus;s__croceus	3	L, LF, BC
Akkermansia;s__muciniphila	3	L, LF, BC
Unclassified;Other	3	L, LF, BC
Actinobaculum;s__massiliense	2	L, LF
Scardovia;s__wiggsiae	2	L, LF
Bacteroides;s__caccae	2	LF, BC
Bacteroides;s__nordii	2	LF, BC
Parabacteroides;s__goldsteinii	2	LF, BC
Bacteroidales;Other	2	LF, BC
Lactobacillus;s__delbrueckii	2	L, BC
Lactobacillus;s__gasseri	2	L, LF
Lactobacillus;s__mucosae	2	L, BC
Leuconostoc;s__	2	L, BC
Leuconostoc;s__lactis	2	L, LF
Leuconostoc;s__mesenteroides	2	L, BC
Streptococcus;s__agalactiae	2	L, LF
Streptococcus;s__anginosus	2	L, LF
Turicibacter;Other	2	L, LF
Alkaliphilus;Other	2	L, LF
Anaerococcus;s__hydrogenalis	2	L, LF
Clostridium;s__nitrophenolicum	2	L, LF
Clostridium;s__paraputrificum	2	L, BC

Clostridium;s__thiosulfatireducens	2	LF, BC
Mogibacterium;s__vescum	2	LF, BC
Peptoniphilus;Other	2	L, LF
Peptoniphilus;s__asaccharolyticus	2	LF, BC
Blautia;s__schinkii	2	L, BC
Blautia;s__wexlerae	2	L, LF
Coprococcus;Other	2	L, LF
Peptostreptococcus;s__stomatis	2	L, LF
Anaerotruncus;s__	2	L, LF
Oscillospira;Other	2	L, BC
Oscillospira;s__guilliermondii	2	L, LF
Acidaminococcus;Other	2	L, BC
Acidaminococcus;s__intestini	2	L, BC
Phascolarctobacterium;s__faecium	2	LF, BC
Veillonella;s__atypica	2	L, LF
Eggerthella;s__sinensis	2	L, BC
Bulleidia;s__extructa	2	L, BC
Fusobacterium;s__	2	L, BC
Fusobacterium;s__nucleatum	2	L, BC
Deltaproteobacteria;Other	2	L, LF
Enterobacter;Other	2	L, BC
Enterobacter;s__amnigenus	2	L, LF
Klebsiella;s__oxytoca	2	LF, BC

Table S11. qPCR absolute quantifications and shifts over time of selected bacterial taxa.

**qPCR Targets & Sample**	**Absolute quantification (cells/mL** ± SD)	**Changes in abundance (Log$_2$(F/C)**		**MANOVA**	**FDR**
	**Baseline = 0 h**	**T1 = 16 h**	**EP = 24 h**	***p* "matrix effect"**	**- Log10(*p*)**
Eubacteria					
L	2.24E+09 ± 7.00E+07	-0.16^{A}	-0.23	0.606265	0.217337
LF	2.24E+09 ± 7.00E+07^{b}	-1.17^{aB}	-0.59^{a}	0.002868	2.542420
BC	2.24E+09 ± 7.00E+07^{b}	-0.51^{aA}	-0.50^{a}	0.025652	1.590878
***p* "time effect"**		0.004994	0.559975		
Firmicutes					
L	2.04E+09 ± 1.57E+07^{b}	-0.89^{aB}	-0.94^{aB}	< 0.000001	6.173925
LF	2.04E+09 ± 1.57E+07^{c}	-1.00^{aB}	-1.26^{bB}	< 0.000001	6.105683
BC	2.04E+09 ± 1.57E+07^{b}	-0.42^{aA}	-0.50^{aA}	0.000455	3.341988
***p* "time effect"**		0.000081	0.000043		
Bacteroidetes					
L	1.47E+08 ± 1.00E+07^{c}	-0.43^{aB}	-1.14^{bC}	0.000002	5.698979
LF	1.47E+08 ± 1.00E+07^{a}	-0.09^{aA}	-0.70^{bB}	0.000008	5.096910
BC	1.47E+08 ± 1.00E+07^{c}	-0.62^{bB}	-0.11^{aA}	0.000199	3.701146
***p* "time effect"**		0.000680	0.000028		

Lactobacillales					
L	7.86E+04 ± 4.74E+03^{c}	0.67^{bA}	1.16^{aA}	< 0.000001	6.346787
LF	7.86E+04 ± 4.74E+03^{c}	0.59^{aB}	-0.25^{bB}	0.000006	5.221848
BC	7.86E+04 ± 4.74E+03^{c}	-0.35^{bC}	-0.20^{aB}	0.000423	3.373659
***p* "time effect"**		0.000003	< 0.000001		
Bifidobacteriaceae					
L	6.15E+05 ± 1.64E+04^{b}	0.49^{a}	0.39^{aA}	0.001150	2.939302
LF	6.15E+05 ± 1.64E+04	0.22	-1.57^{B}	0.151903	0.818433
BC	6.15E+05 ± 1.64E+04^{a}	0.04^{a}	-2.56^{bC}	0.000033	4.48148
***p* "time effect"**		0.261793	0.001231		
Enterobacteriaceae					
L	2.38E+05 ± 7.60E+03^{ab}	2.33^{a}	2.37^{bB}	0.000034	4.468521
LF	2.38E+05 ± 7.60E+03c	5.59^{b}	7.71^{aA}	0.000002	5.698976
BC	2.38E+05 ± 7.60E+03^{b}	5.76^{b}	7.94^{aA}	< 0.000001	6.071911
***p* "time effect"**		0.760759	0.000001		

^{A,B,C} Different capital letters indicate significance difference within a column; ^{a,b,c} Different lower case letters indicate statistical significance within a row according to ANOVA model followed by Tukey's HSD test (p < 0.05). MANOVA p values are relative to "time effect" on rows and to "matrix effect" on columns; FDR = False Discovery Rate as -Log10(p) indicates significance of Log_2(F/C); L = Standard milk; LF = Lactose-free milk; BC = Blank control; BL = Baseline; T1 = 16 h of fermentation; EP = 24 h of fermentation.

Supplementary References

Lane, D.J., Harrison, Jr. A. P., Stahl, D., Pace, B., Giovannoni, S. J., Olsen, G. J., Pace, N. R. (1992). Evolutionary relationships among sulfur- and iron-oxidizing eubacteria. *Journal of Bacteriology*, 174(1), 269-278.

Guo, X., Xia, X., Tang, R., Zhou, J., Zhao, H., Wang, K. (2008). Development of a real-time PCR method for *Firmicutes* and *Bacteroidetes* in faeces and its application to quantify intestinal population of obese and lean pigs. *Letters in Applied Microbiology*, 47(5), 367-73.

Walter, J., Hertel, C., Tannock, G. W., Lis, C. M., Munro, K., Hammes, W. P. (2001). Detection of *Lactobacillus, Pediococcus, Leuconostoc,* and *Weissella* species in human feces by using group-specific PCR primers and Denaturing Gradient Gel Electrophoresis. *Applied and Environmental Microbiology,* 67(6), 2578-2585.

Masco, L., Ventura, M., Zink, R., X Huys, J., Swings, V. (2004). Polyphasic taxonomic analysis of *Bifidobacterium animalis* and *Bifidobacterium lactis* reveals relatedness at the subspecies level: reclassification of *Bifidobacterium animalis* as *Bifidobacterium animalis subsp. animalis subsp. nov.* and *Bifidobacterium lactis* as *Bifidobacterium animalis subsp. lactis subsp. nov., International Journal of Systematic and. Evolutionary Microbiology*, 54, 1137–1143.

Bartosch, S., Fite, A., Macfarlane, G. T., McMurdo, M. E. (2004). Characterization of bacterial communities in feces from healthy elderly volunteers and hospitalized elderly patients by using real-time PCR and effects of antibiotic treatment on the fecal microbiota, *Applied and Environmental Microbiology*, 70(6), 3575-81.
